# Supplementary material for: A Humanized Yeast Phenomic Model of Deoxycytidine Kinase to Predict Genetic Buffering of Nucleoside Analog Cytotoxicity
Source: Genes (Basel). 2019 Sep 30;10(10):770. doi: 10.3390/genes10100770 (PMC6826991; doi:10.3390/genes10100770)
Supplement: Supplementary file 1 [file genes-10-00770-s001.zip › Additional_Files/Additional_File7_GOTermHeatmaps.zip!/Additional_File7_GOTermHeatmaps/transcription_factor_activity,_transcription_factor_binding.pdf]

transcription factor activity, transcription factor binding

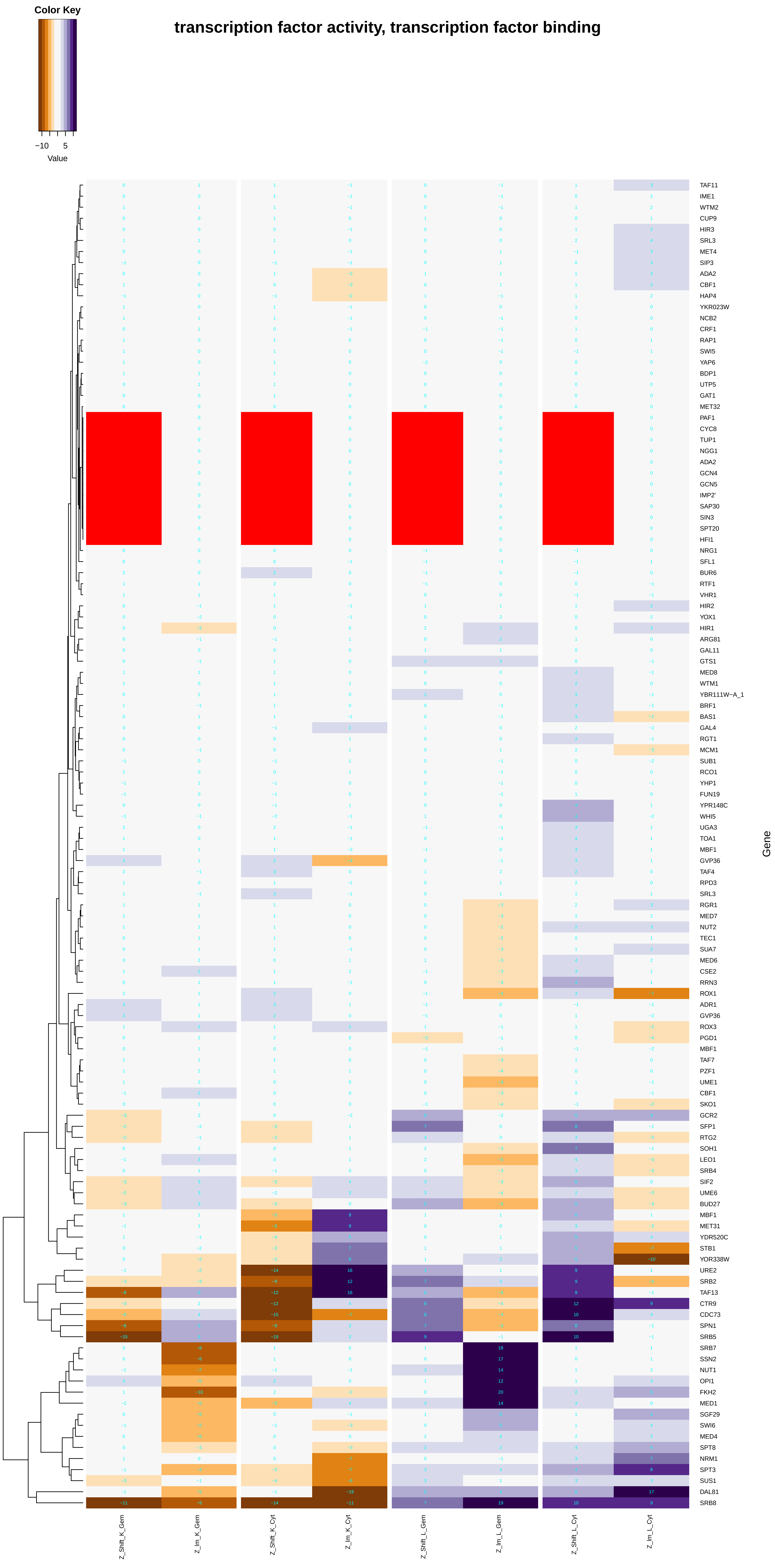

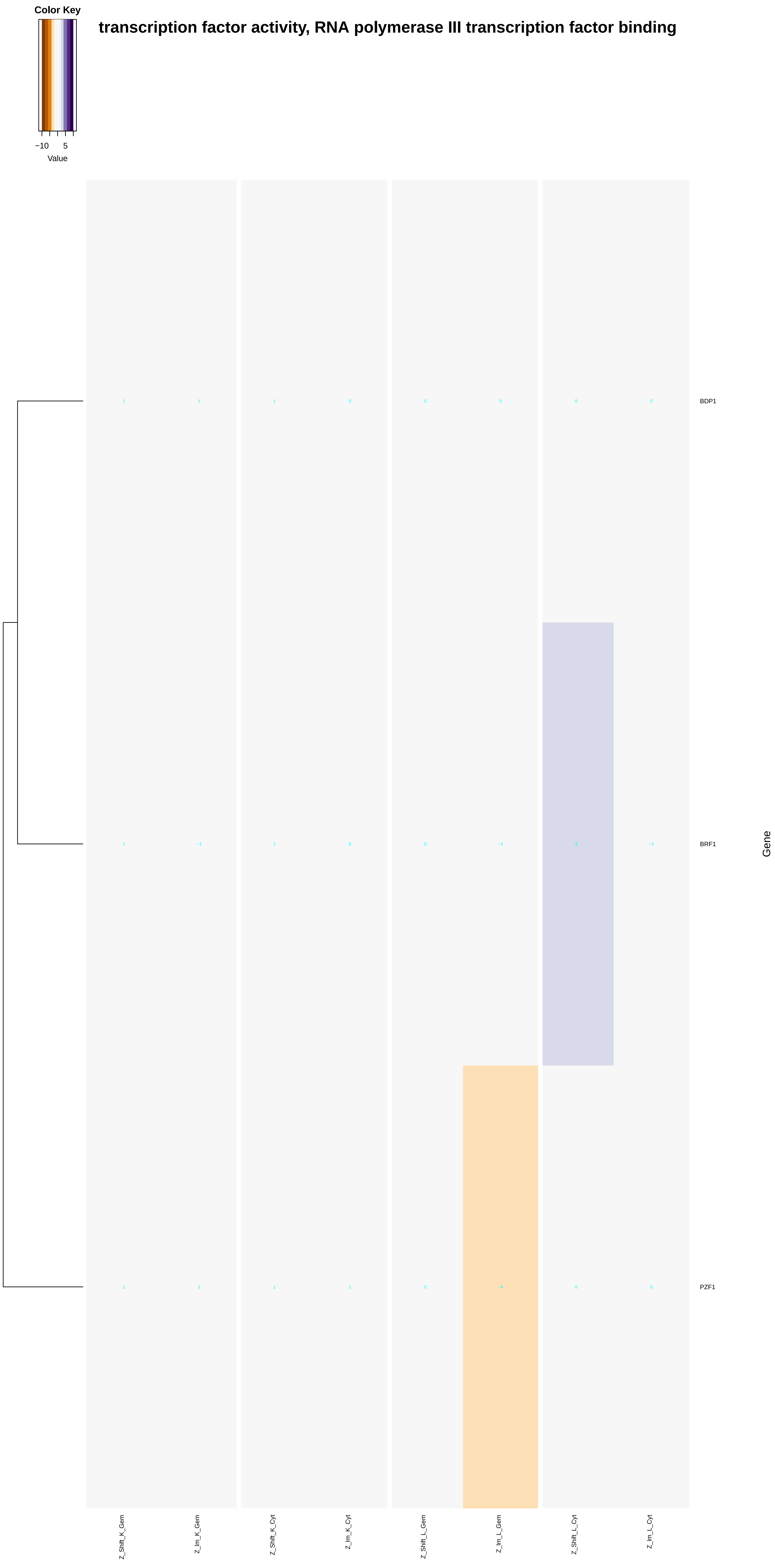

Color Key
−10
5
Value
transcription factor activity, RNA polymerase III transcription factor binding
BDP1
BRF1
PZF1
Gene
Z_Shift_K_Gem
Z_Im_K_Gem
Z_Shift_K_Cyt
Z_Im_K_Cyt
Z_Shift_L_Gem
Z_Im_L_Gem
Z_Shift_L_Cyt
Z_Im_L_Cyt

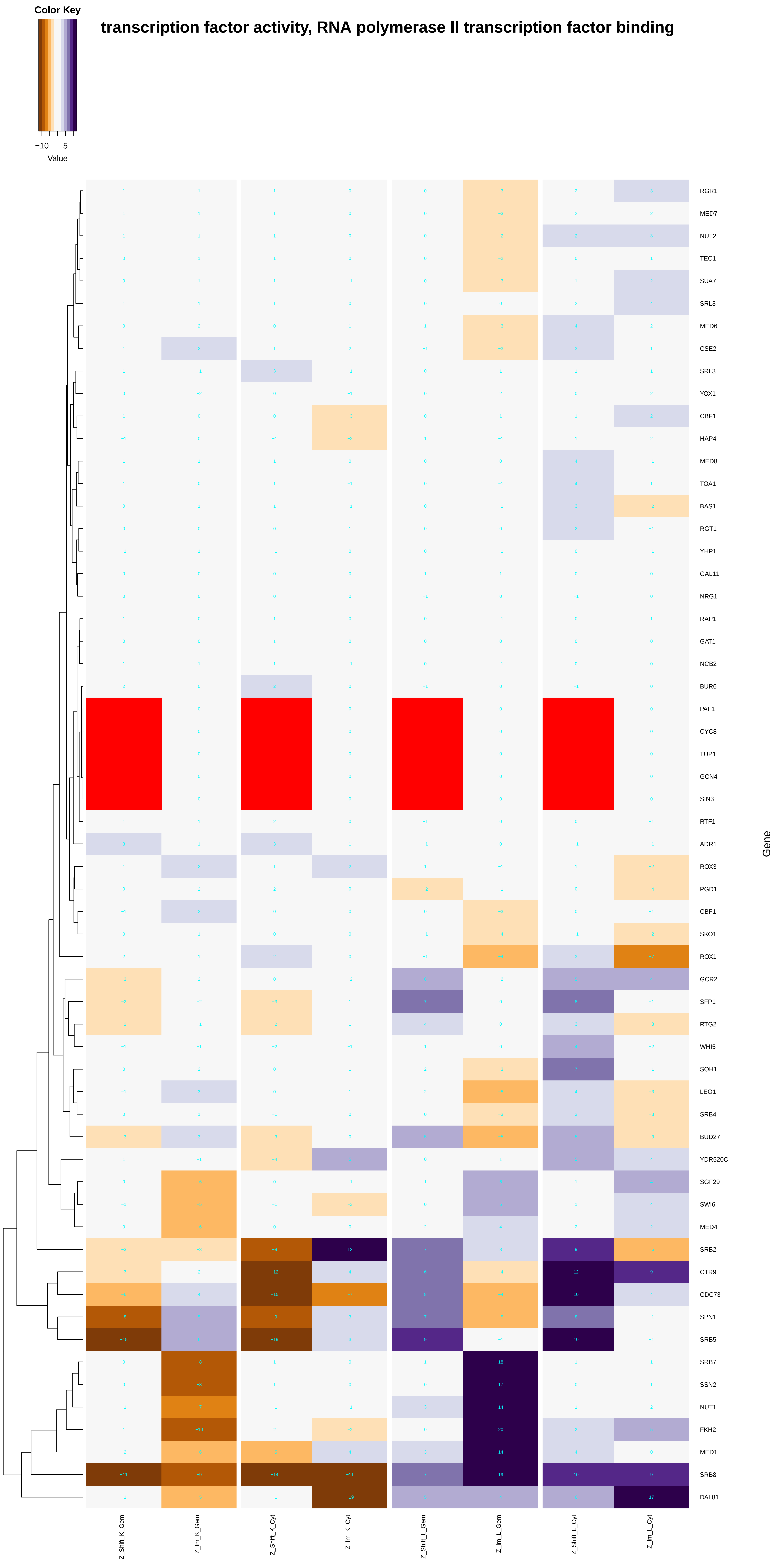

Color Key
−10
5
Value
transcription factor activity, RNA polymerase II transcription factor binding
RGR1
MED7
NUT2
TEC1
SUA7
SRL3
MED6
CSE2
SRL3
YOX1
CBF1
HAP4
MED8
TOA1
BAS1
RGT1
YHP1
GAL11
NRG1
RAP1
GAT1
NCB2
BUR6
PAF1
CYC8
TUP1
GCN4
SIN3
RTF1
ADR1
ROX3
PGD1
CBF1
SKO1
ROX1
GCR2
SFP1
RTG2
WHI5
SOH1
LEO1
SRB4
BUD27
YDR520C
SGF29
SWI6
MED4
SRB2
CTR9
CDC73
SPN1
SRB5
SRB7
SSN2
NUT1
FKH2
MED1
SRB8
DAL81
Gene
Z_Shift_K_Gem
Z_Im_K_Gem
Z_Shift_K_Cyt
Z_Im_K_Cyt
Z_Shift_L_Gem
Z_Im_L_Gem
Z_Shift_L_Cyt
Z_Im_L_Cyt

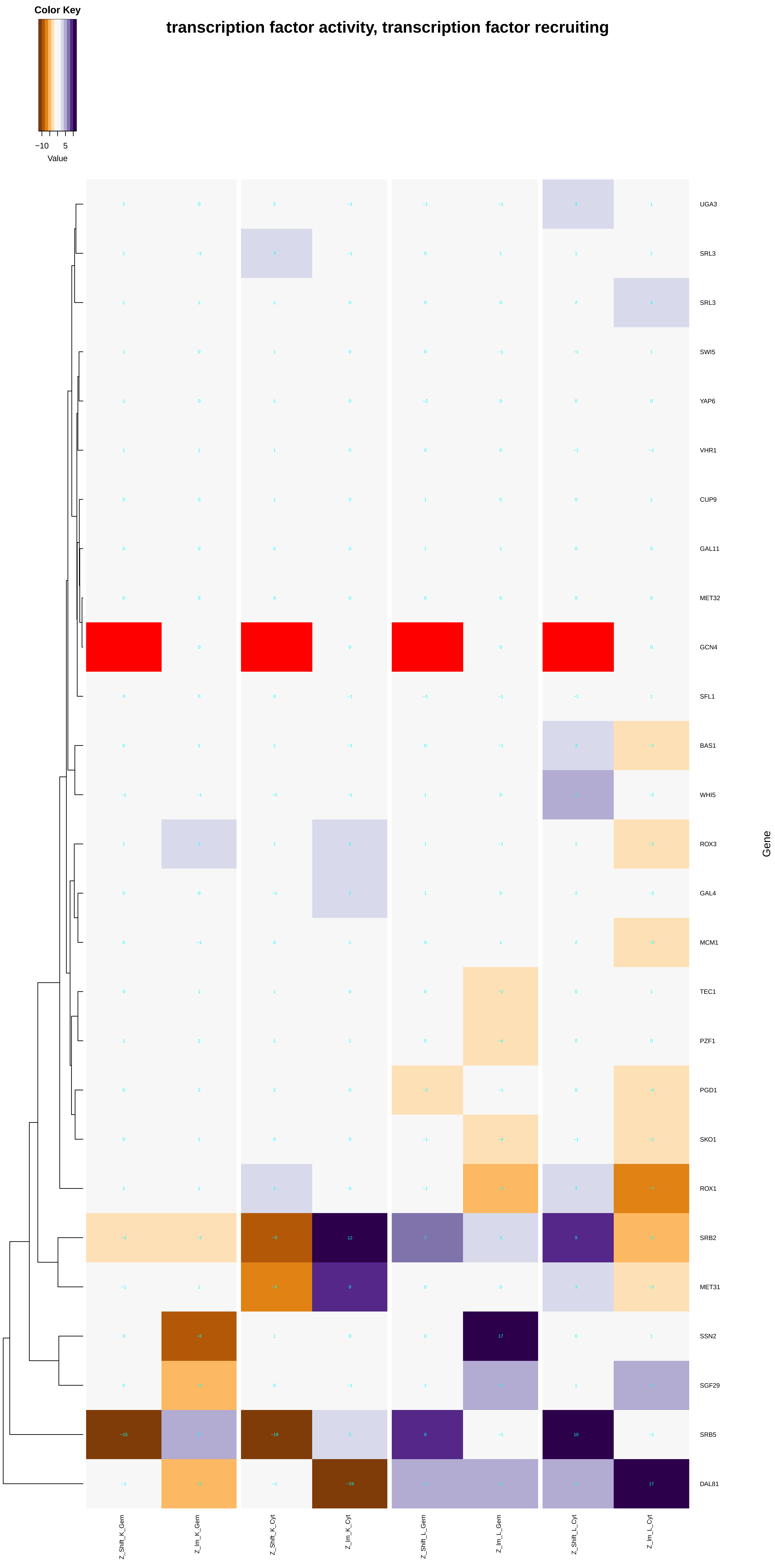
transcription factor activity, transcription factor recruiting
Color Key
−10
5
Value
UGA3
SRL3
SRL3
SWI5
YAP6
VHR1
CUP9
GAL11
MET32
GCN4
SFL1
BAS1
WHI5
ROX3
GAL4
MCM1
TEC1
PZF1
PGD1
SKO1
ROX1
SRB2
MET31
SSN2
SGF29
SRB5
DAL81
Gene
Z_Shift_K_Gem
Z_Im_K_Gem
Z_Shift_K_Cyt
Z_Im_K_Cyt
Z_Shift_L_Gem
Z_Im_L_Gem
Z_Shift_L_Cyt
Z_Im_L_Cyt

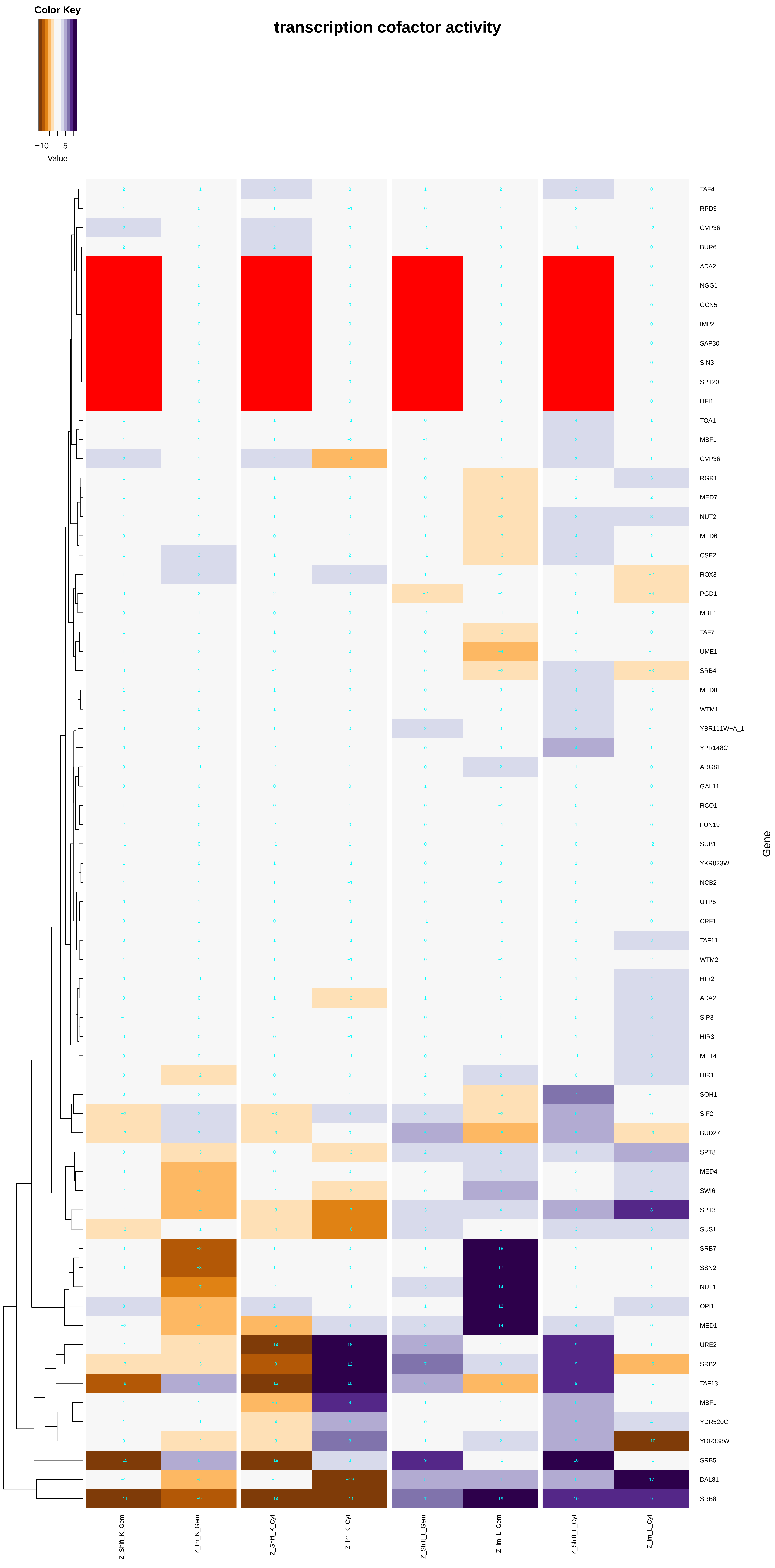
Color Key
−10
5
Value
transcription cofactor activity
TAF4
RPD3
GVP36
BUR6
ADA2
NGG1
GCN5
IMP2'
SAP30
SIN3
SPT20
HFI1
TOA1
MBF1
GVP36
RGR1
MED7
NUT2
MED6
CSE2
ROX3
PGD1
MBF1
TAF7
UME1
SRB4
MED8
WTM1
YBR111W−A_1
YPR148C
ARG81
GAL11
RCO1
FUN19
SUB1
YKR023W
NCB2
UTP5
CRF1
TAF11
WTM2
HIR2
ADA2
SIP3
HIR3
MET4
HIR1
SOH1
SIF2
BUD27
SPT8
MED4
SWI6
SPT3
SUS1
SRB7
SSN2
NUT1
OPI1
MED1
URE2
SRB2
TAF13
MBF1
YDR520C
YOR338W
SRB5
DAL81
SRB8
Gene
Z_Shift_K_Gem
Z_Im_K_Gem
Z_Shift_K_Cyt
Z_Im_K_Cyt
Z_Shift_L_Gem
Z_Im_L_Gem
Z_Shift_L_Cyt
Z_Im_L_Cyt

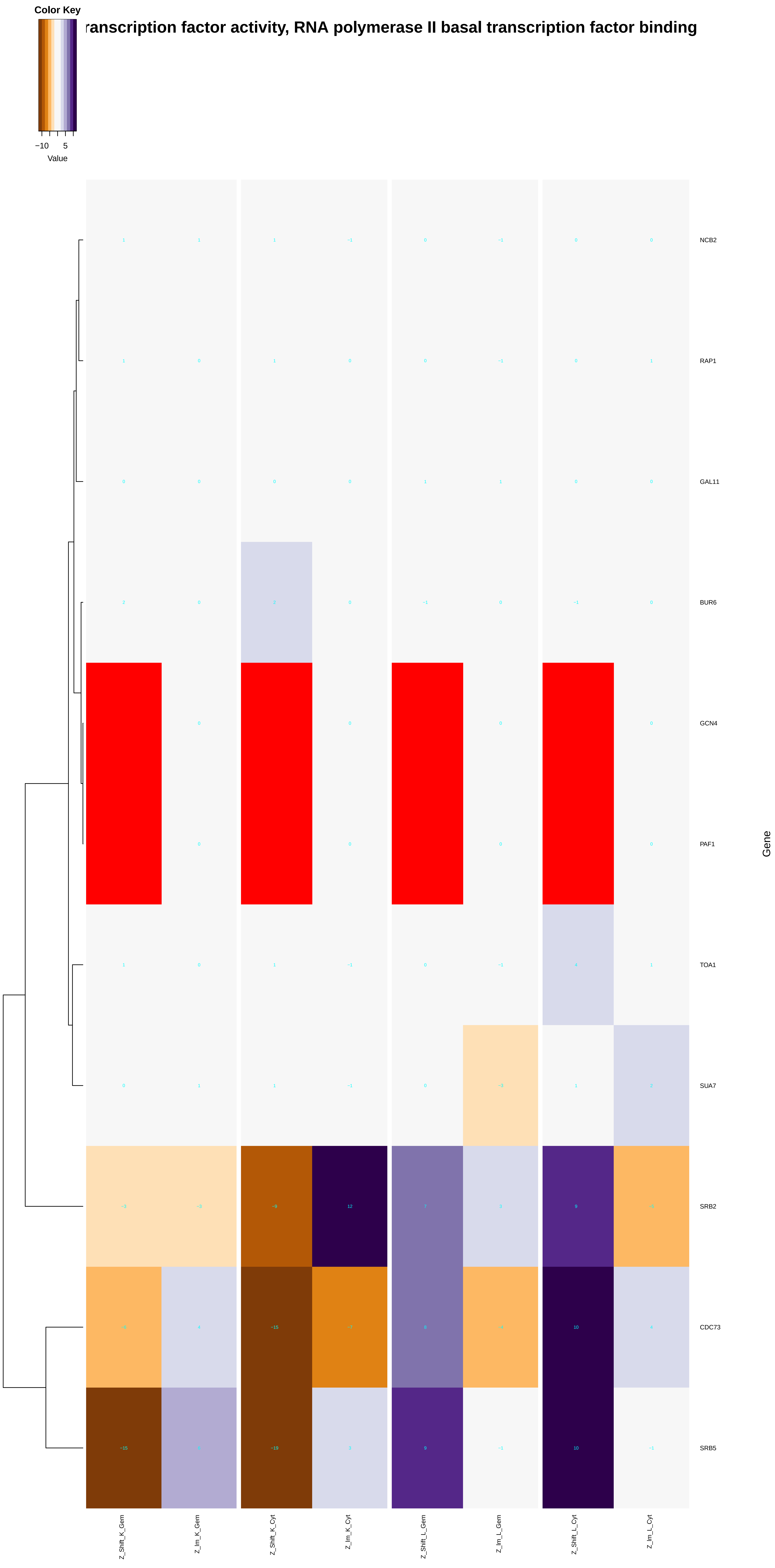

Color Key
−10
5
Value
ranscription factor activity, RNA polymerase II basal transcription factor binding
NCB2
RAP1
GAL11
BUR6
GCN4
PAF1
TOA1
SUA7
SRB2
CDC73
SRB5
Gene
Z_Shift_K_Gem
Z_Im_K_Gem
Z_Shift_K_Cyt
Z_Im_K_Cyt
Z_Shift_L_Gem
Z_Im_L_Gem
Z_Shift_L_Cyt
Z_Im_L_Cyt

RNA polymerase II transcription cofactor activity

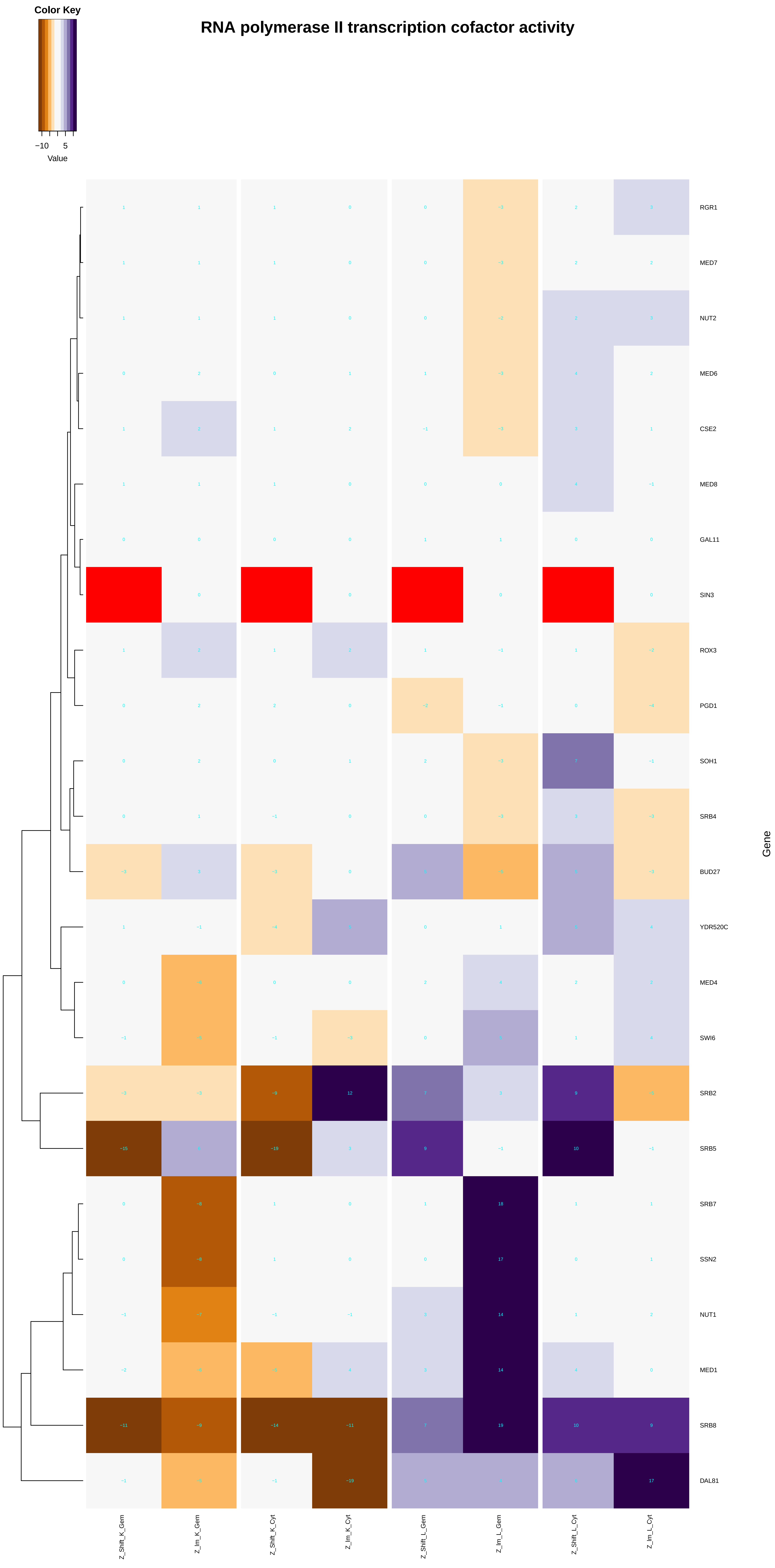

transcription factor activity, RNA polymerase II transcription factor recruiting

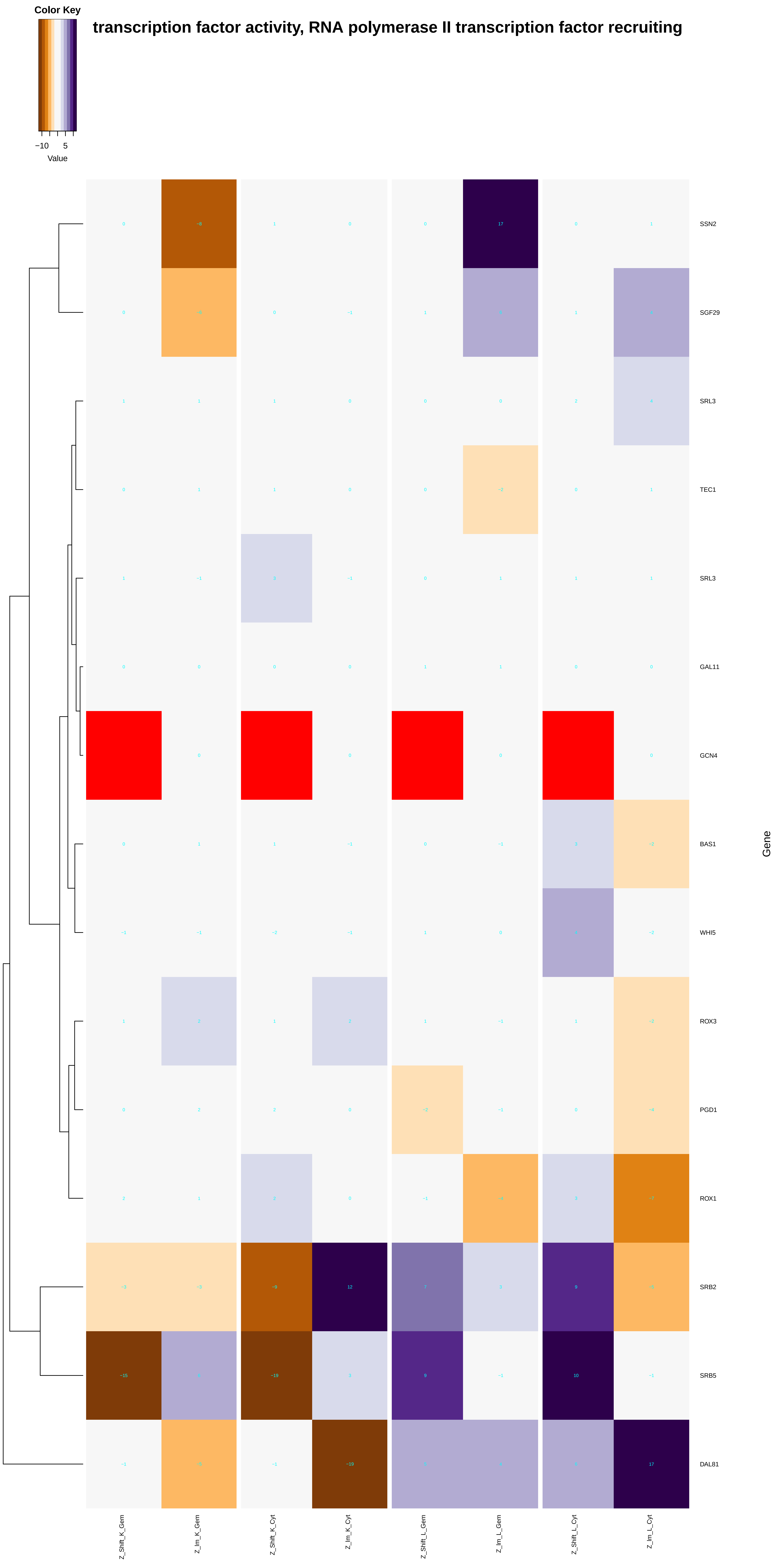

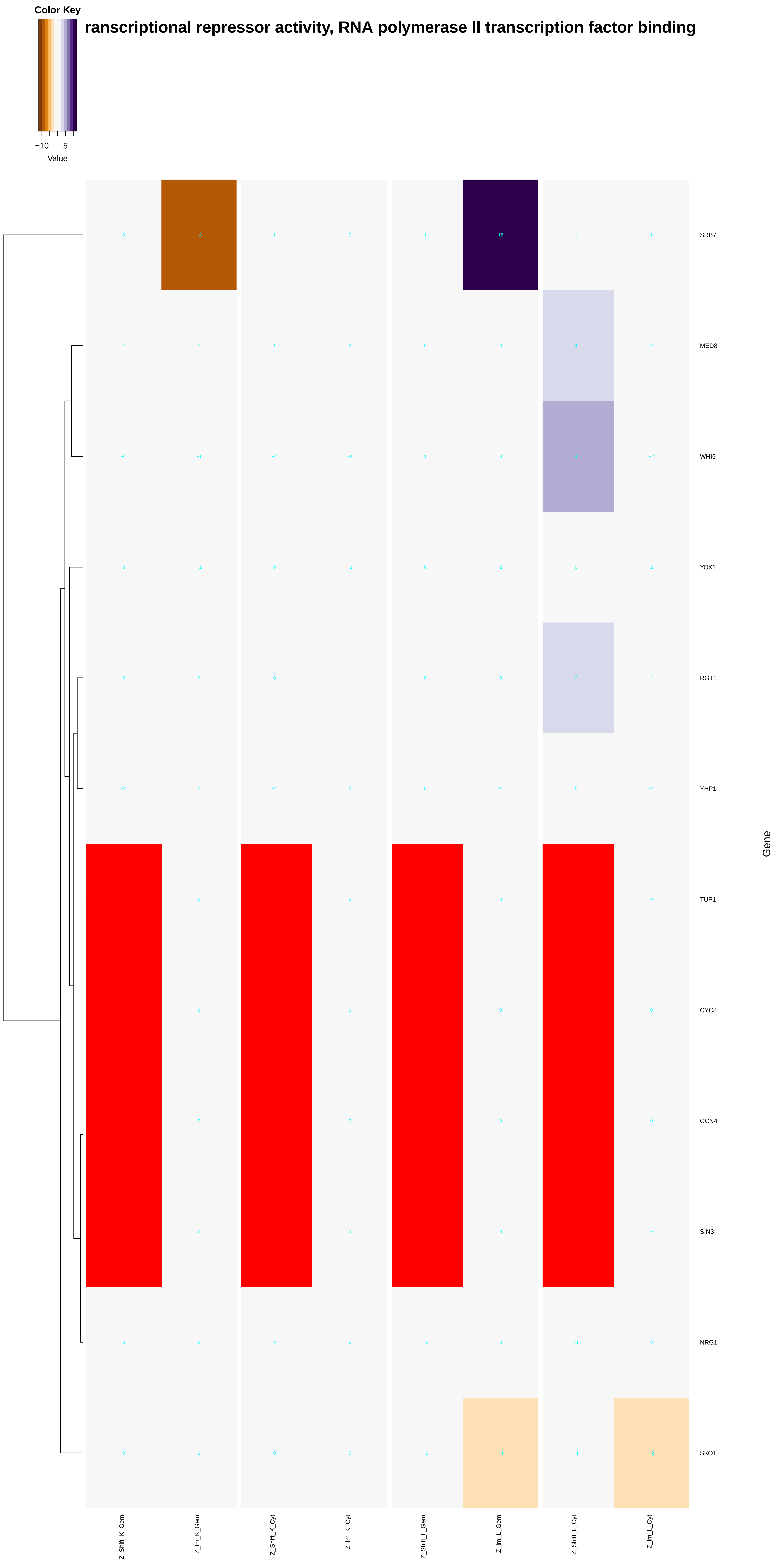

Color Key
−10
5
Value
ranscriptional repressor activity, RNA polymerase II transcription factor binding
SRB7
MED8
WHI5
YOX1
RGT1
YHP1
TUP1
CYC8
GCN4
SIN3
NRG1
SKO1
Gene
Z_Shift_K_Gem
Z_Im_K_Gem
Z_Shift_K_Cyt
Z_Im_K_Cyt
Z_Shift_L_Gem
Z_Im_L_Gem
Z_Shift_L_Cyt
Z_Im_L_Cyt

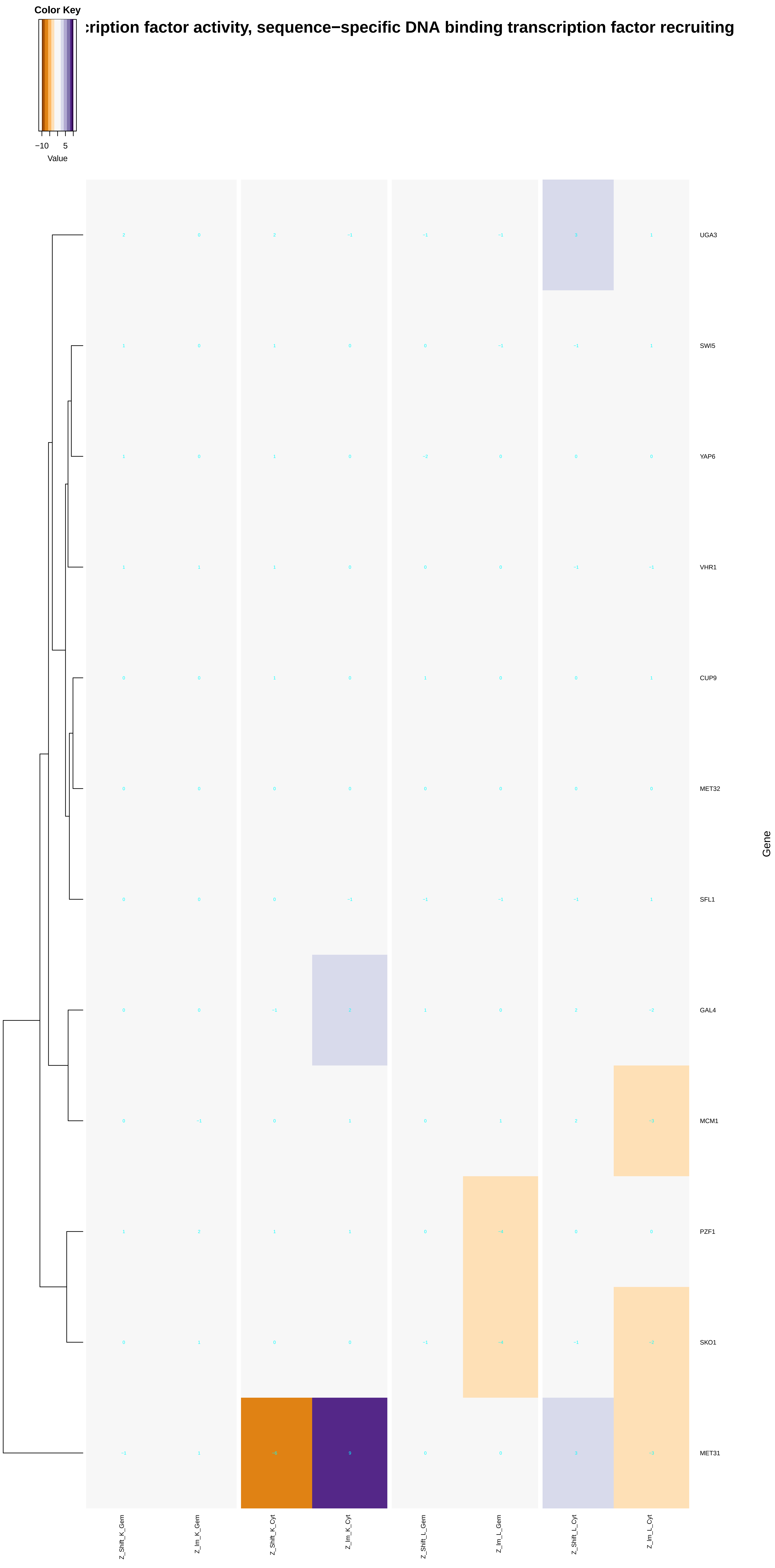

Color Key
−10
5
Value
cription factor activity, sequence−specific DNA binding transcription factor recruiting
UGA3
SWI5
YAP6
VHR1
CUP9
MET32
SFL1
GAL4
MCM1
PZF1
SKO1
MET31
Gene
Z_Shift_K_Gem
Z_lm_K_Gem
Z_Shift_K_Cyt
Z_lm_K_Cyt
Z_Shift_L_Gem
Z_lm_L_Gem
Z_Shift_L_Cyt
Z_lm_L_Cyt

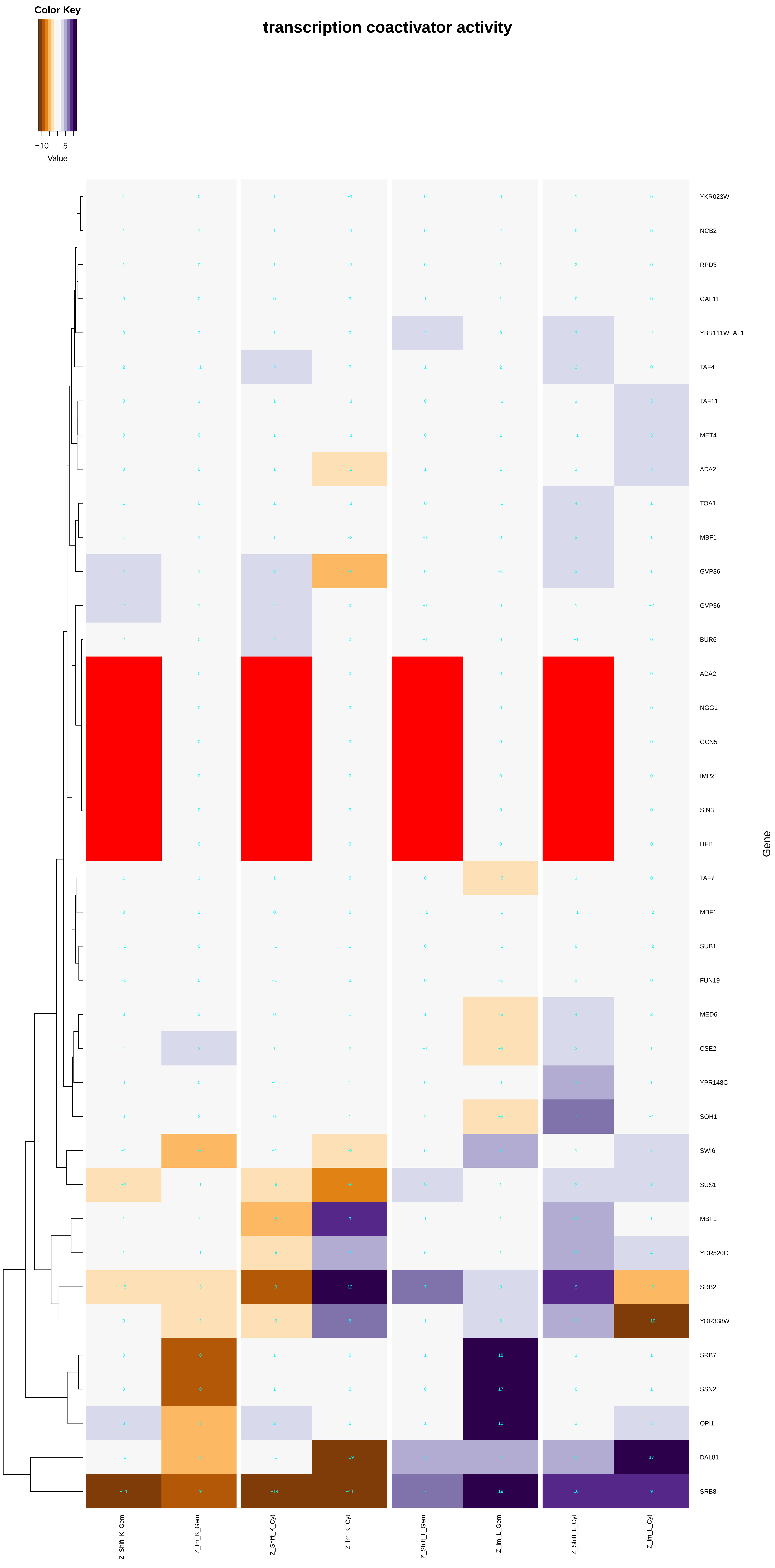
Color Key
−10
5
Value
transcription coactivator activity
YKR023W
NCB2
RPD3
GAL11
YBR111W−A_1
TAF4
TAF11
MET4
ADA2
TOA1
MBF1
GVP36
GVP36
BUR6
ADA2
NGG1
GCN5
IMP2'
SIN3
HFI1
TAF7
MBF1
SUB1
FUN19
MED6
CSE2
YPR148C
SOH1
SWI6
SUS1
MBF1
YDR520C
SRB2
YOR338W
SRB7
SSN2
OPI1
DAL81
SRB8
Gene
Z_Shift_K_Gem
Z_lm_K_Gem
Z_Shift_K_Cyt
Z_lm_K_Cyt
Z_Shift_L_Gem
Z_lm_L_Gem
Z_Shift_L_Cyt
Z_lm_L_Cyt

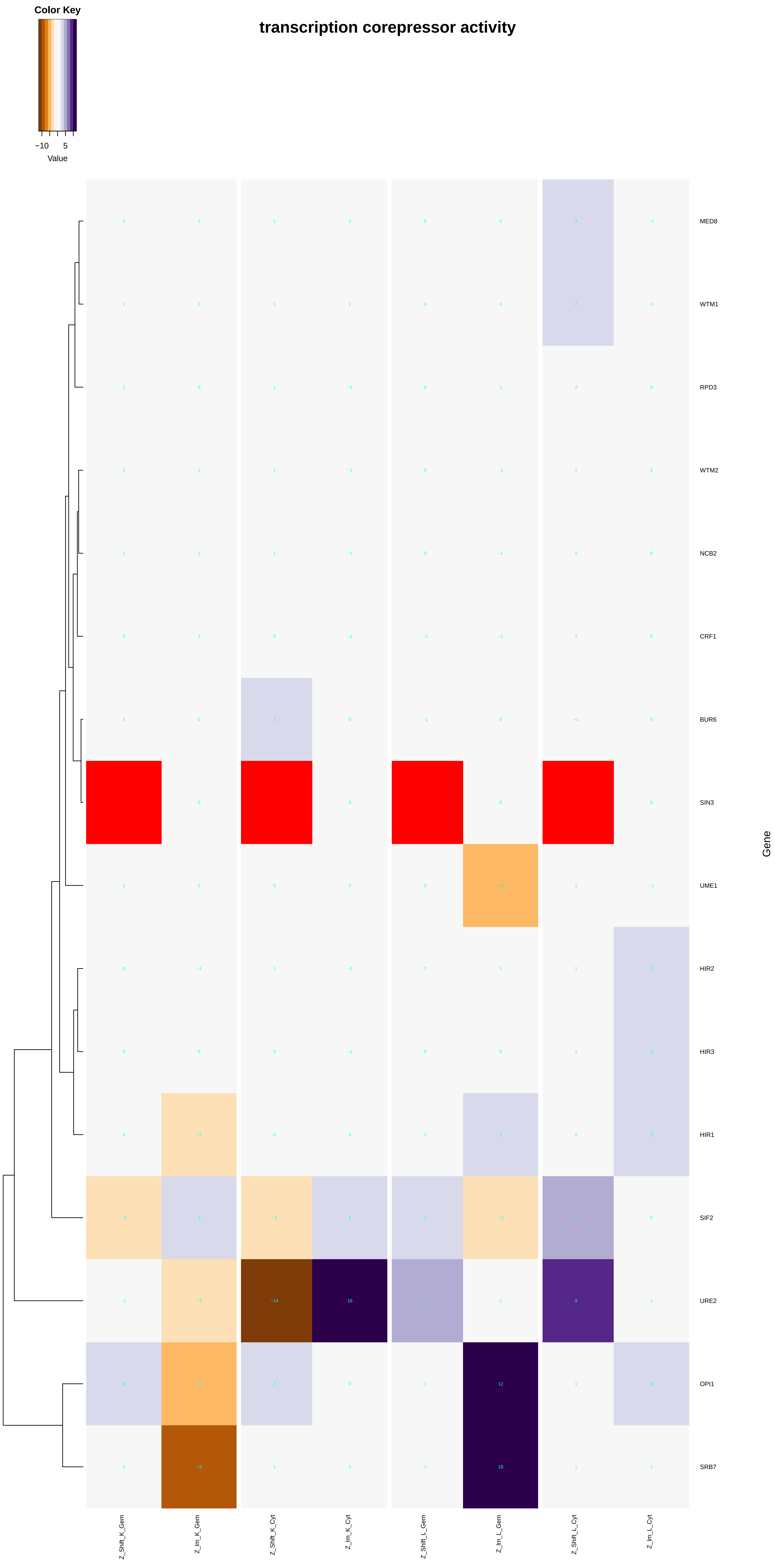
Color Key
−10
5
Value
transcription corepressor activity
MED8
WTM1
RPD3
WTM2
NCB2
CRF1
BUR6
SIN3
UME1
HIR2
HIR3
HIR1
SIF2
URE2
OPI1
SRB7
Gene
Z_Shift_K_Gem
Z_Im_K_Gem
Z_Shift_K_Cyt
Z_Im_K_Cyt
Z_Shift_L_Gem
Z_Im_L_Gem
Z_Shift_L_Cyt
Z_Im_L_Cyt

RNA polymerase II transcription factor activity, TBP−class protein binding

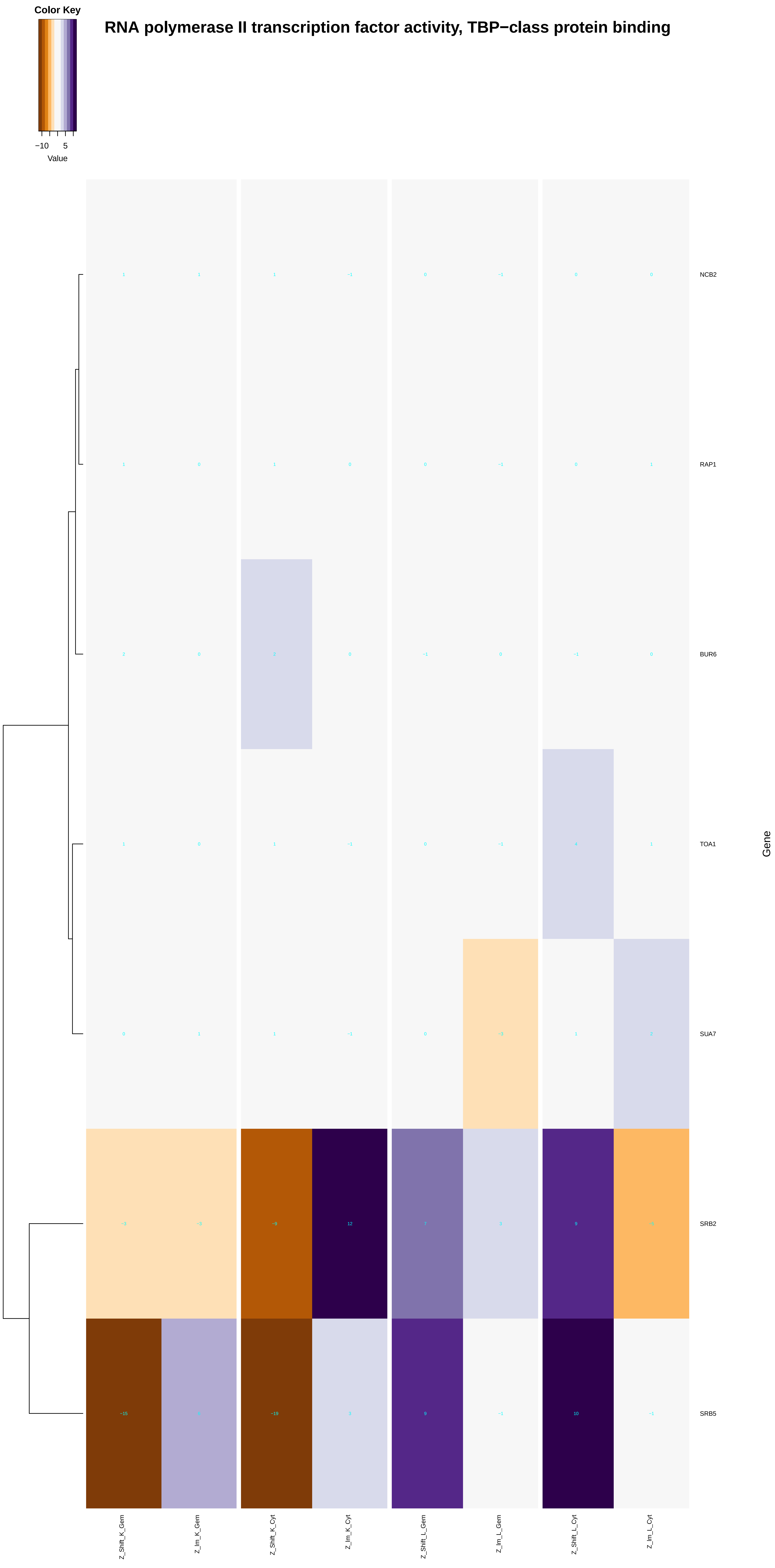

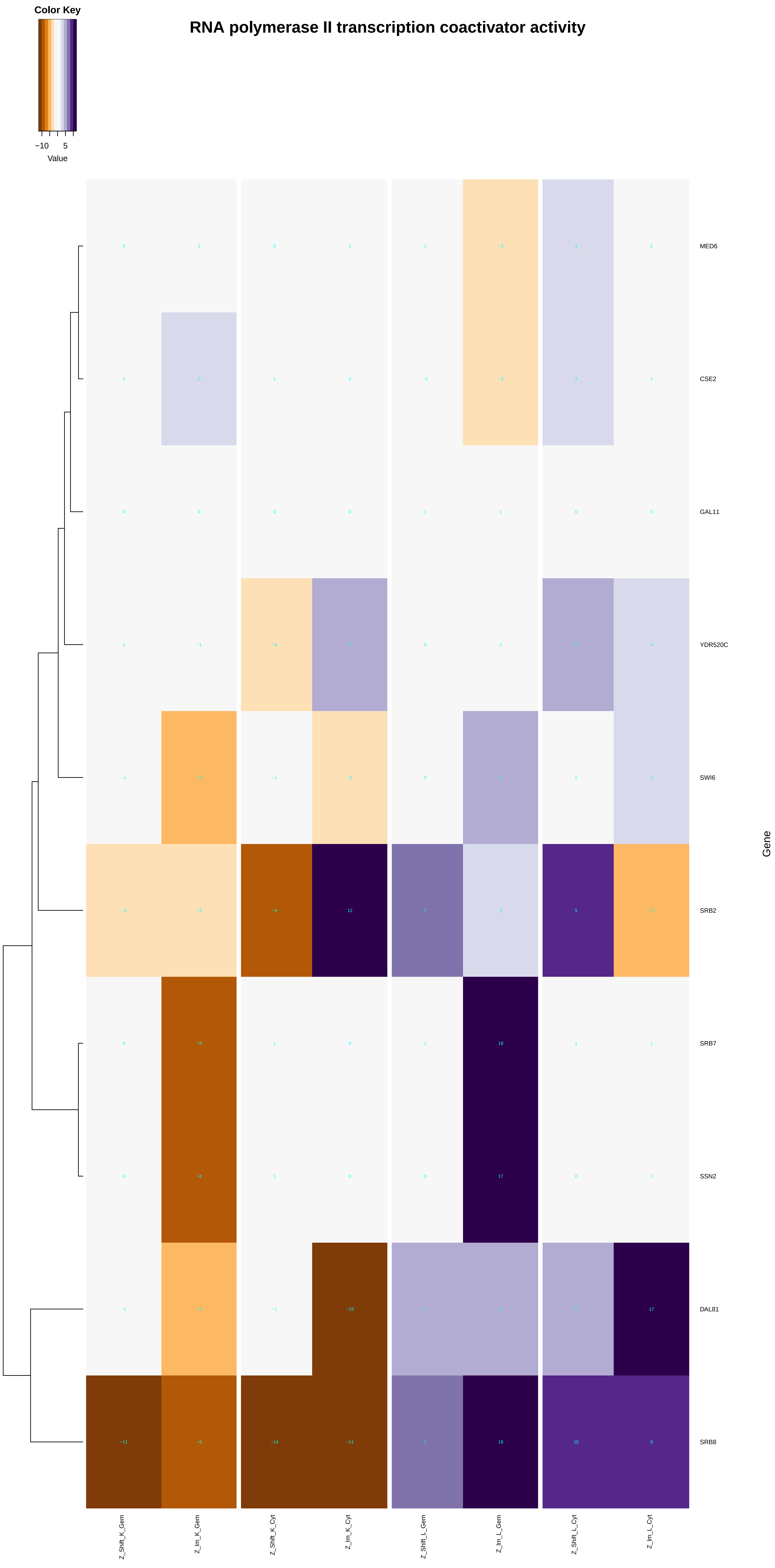

RNA polymerase II transcription coactivator activity
Color Key
−10
5
Value
MED6
CSE2
GAL11
YDR520C
SWI6
SRB2
SRB7
SSN2
DAL81
SRB8
Gene
Z_Shift_K_Gem
Z_Im_K_Gem
Z_Shift_K_Cyt
Z_Im_K_Cyt
Z_Shift_L_Gem
Z_Im_L_Gem
Z_Shift_L_Cyt
Z_Im_L_Cyt

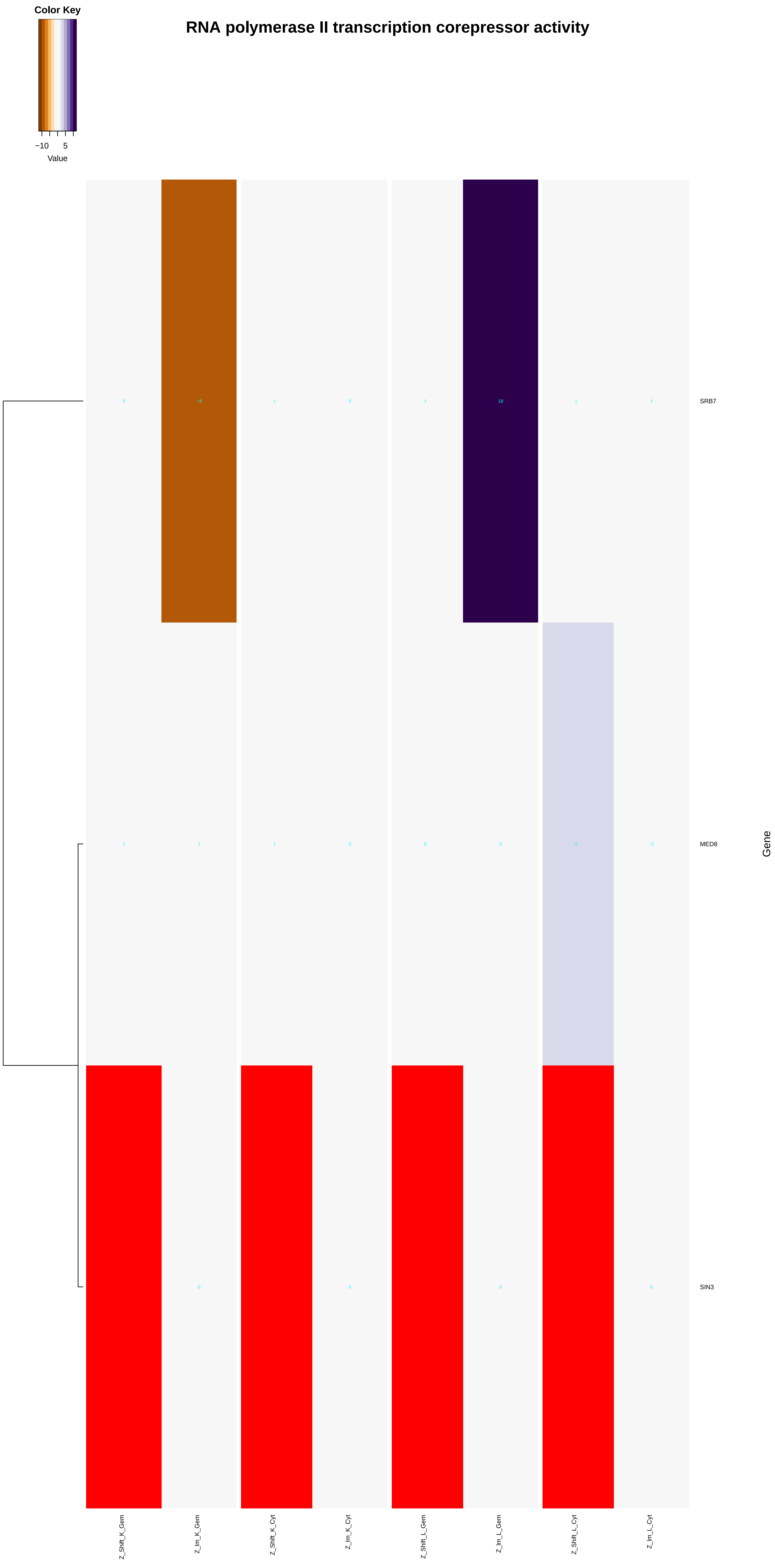
Color Key
−10
5
Value
RNA polymerase II transcription corepressor activity
0
−8
1
0
1
18
1
1
SRB7
1
1
1
0
0
0
4
−1
MED8
0
0
0
0
SIN3
Gene
Z_Shift_K_Gem
Z_Im_K_Gem
Z_Shift_K_Cyt
Z_Im_K_Cyt
Z_Shift_L_Gem
Z_Im_L_Gem
Z_Shift_L_Cyt
Z_Im_L_Cyt

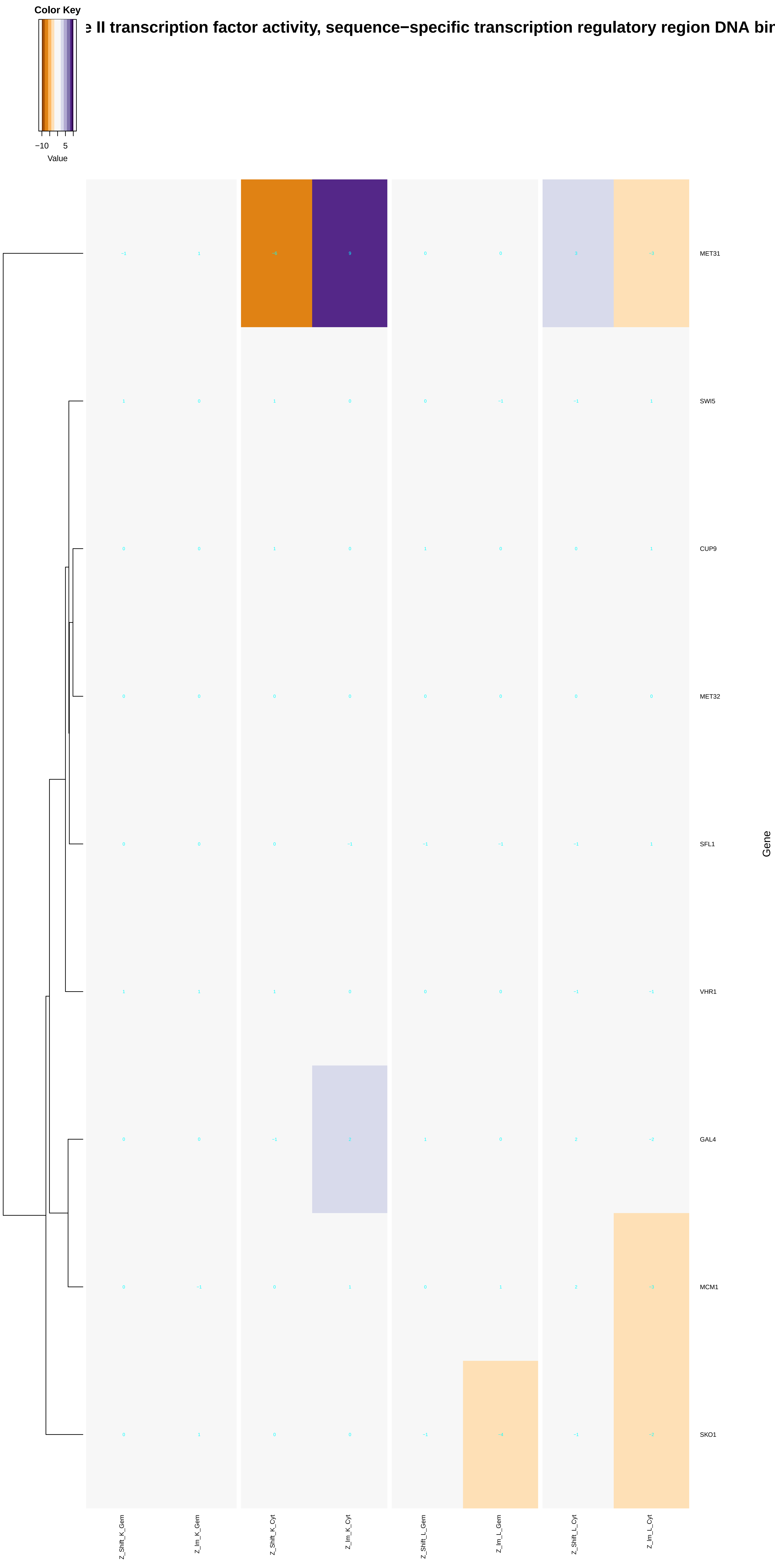

Color Key
−10
5
Value
e II transcription factor activity, sequence−specific transcription regulatory region DNA bir
MET31
SWI5
CUP9
MET32
SFL1
VHR1
GAL4
MCM1
SKO1
Gene
Z_Shift_K_Gem
Z_Im_K_Gem
Z_Shift_K_Cyt
Z_Im_K_Cyt
Z_Shift_L_Gem
Z_Im_L_Gem
Z_Shift_L_Cyt
Z_Im_L_Cyt

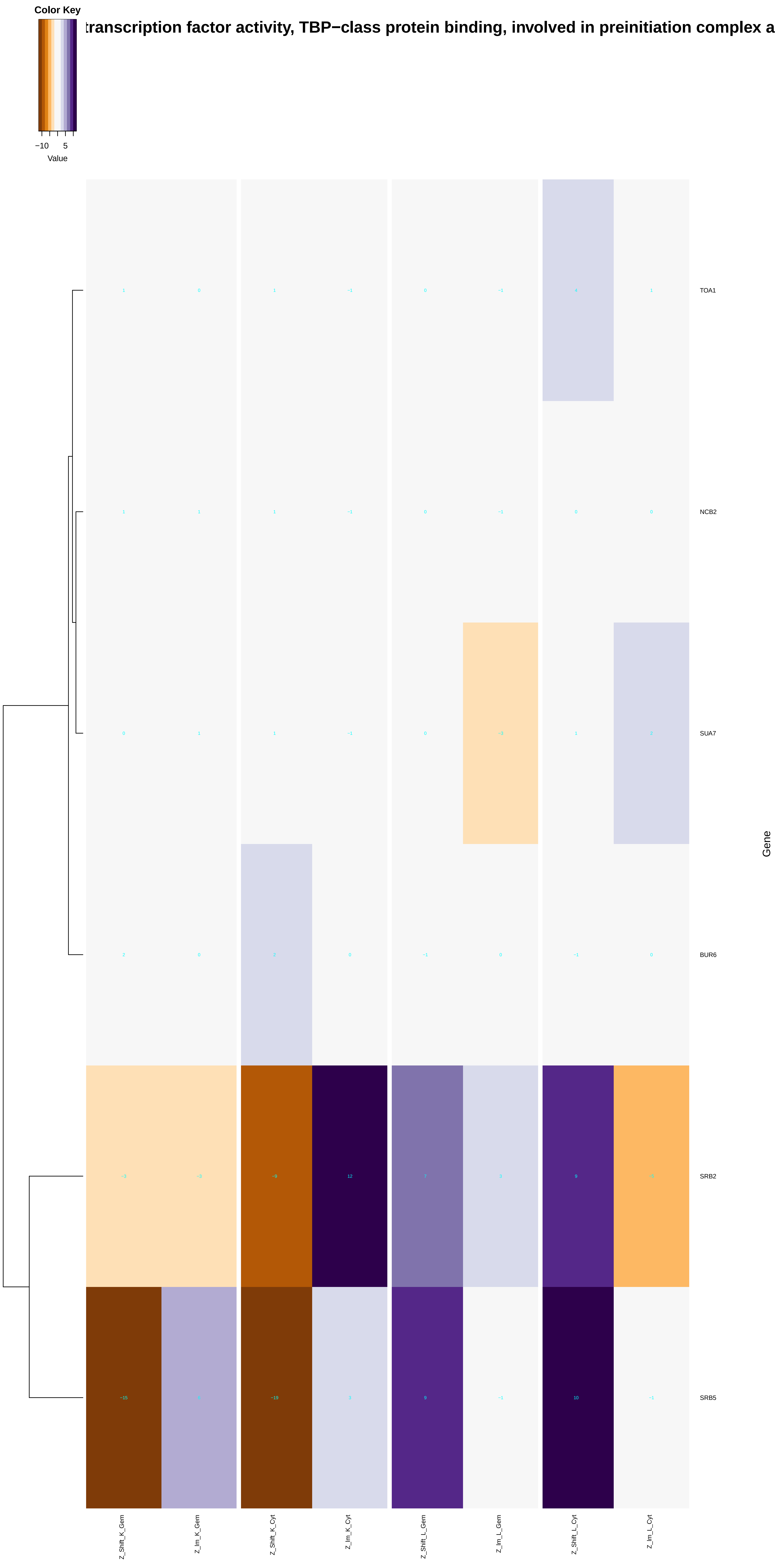

transcription factor activity, TBP−class protein binding, involved in preinitiation complex a
Color Key
−10
5
Value
TOA1
NCB2
SUA7
BUR6
SRB2
SRB5
Gene
Z_Shift_K_Gem
Z_Im_K_Gem
Z_Shift_K_Cyt
Z_Im_K_Cyt
Z_Shift_L_Gem
Z_Im_L_Gem
Z_Shift_L_Cyt
Z_Im_L_Cyt

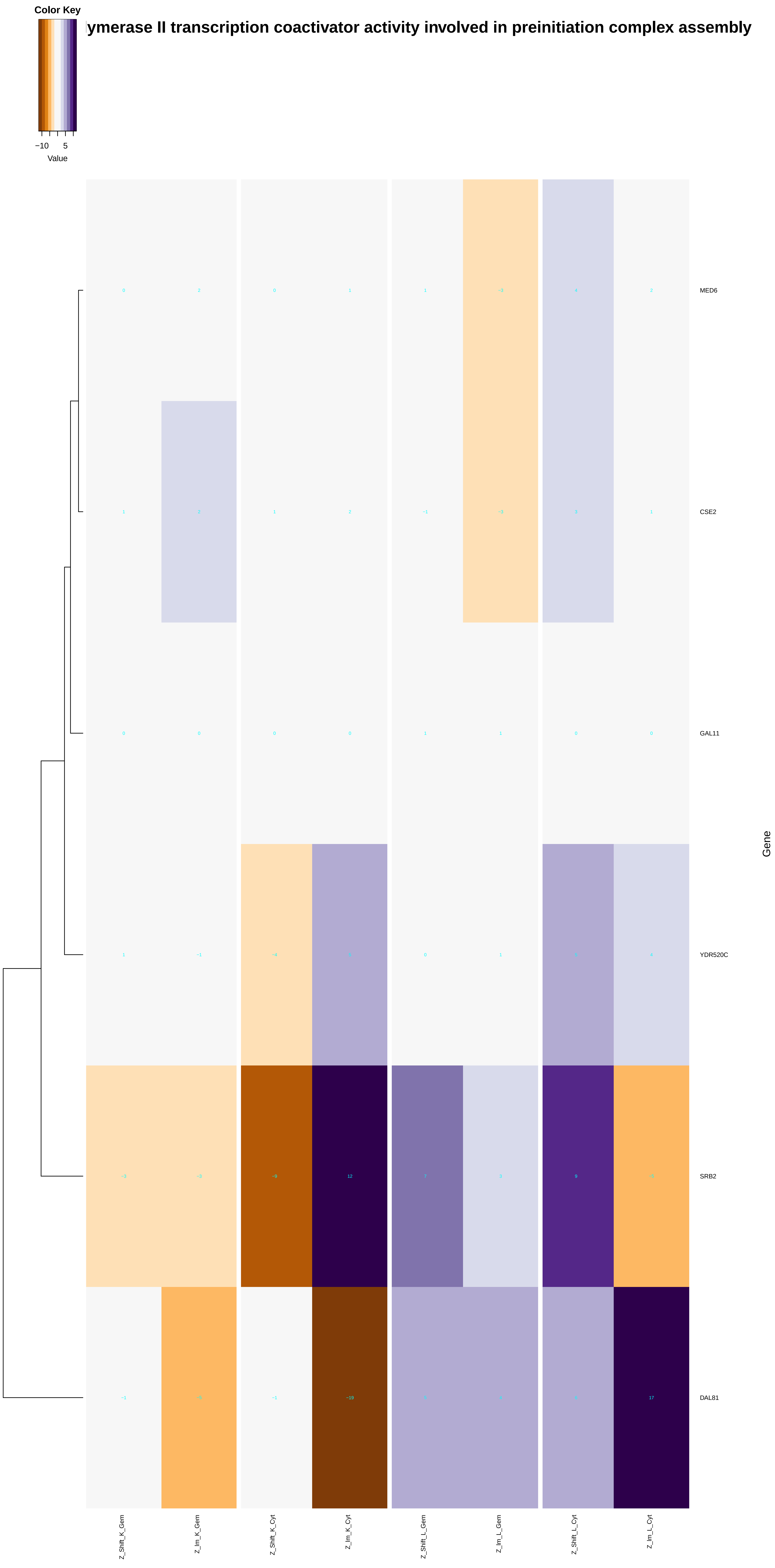
Color Key
−10 5
Value
lymerase II transcription coactivator activity involved in preinitiation complex assembly
MED6
CSE2
GAL11
YDR520C
SRB2
DAL81
Gene
Z_Shift_K_Gem
Z_Im_K_Gem
Z_Shift_K_Cyt
Z_Im_K_Cyt
Z_Shift_L_Gem
Z_Im_L_Gem
Z_Shift_L_Cyt
Z_Im_L_Cyt

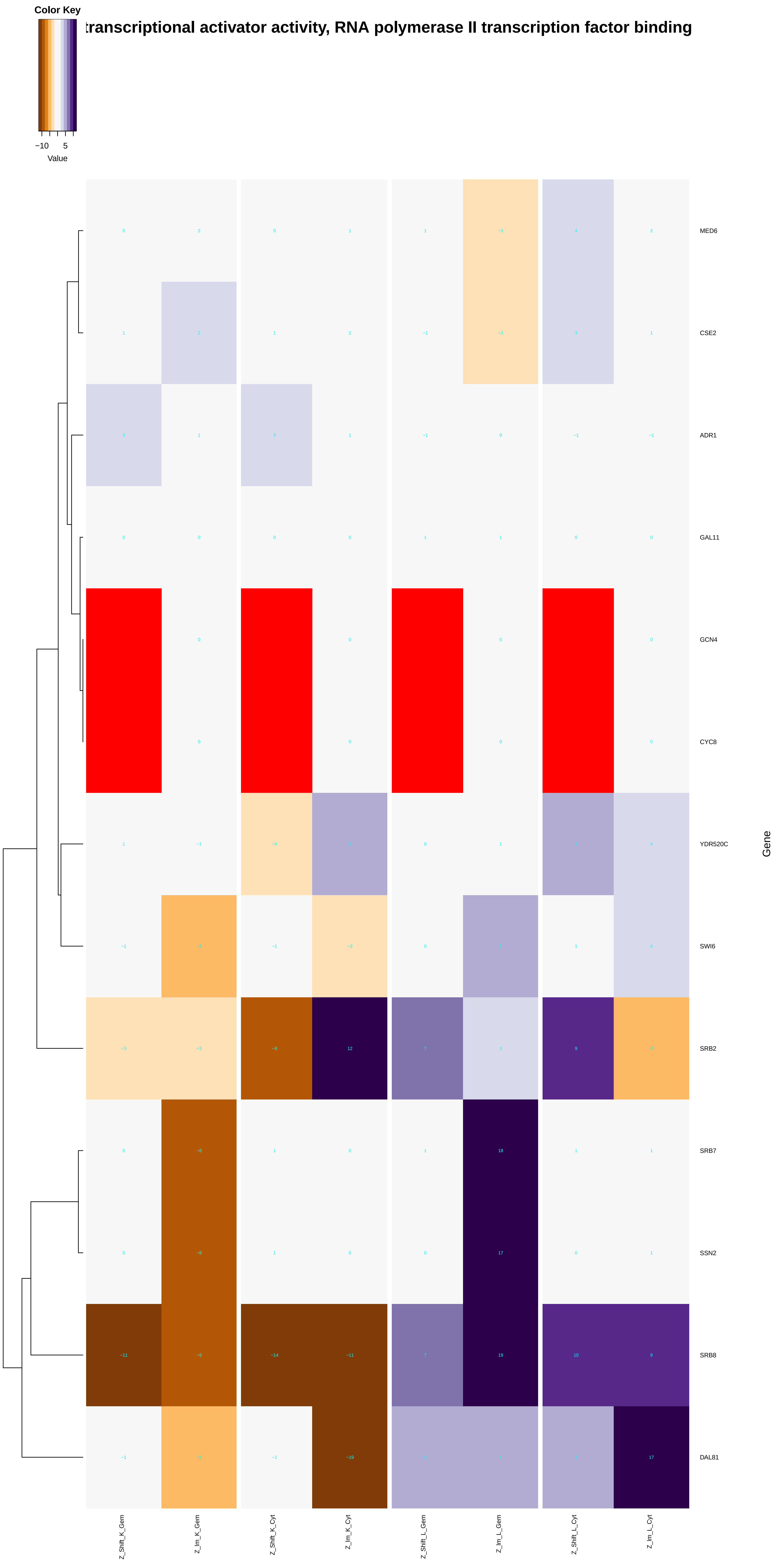

Color Key
−10
5
Value
transcriptional activator activity, RNA polymerase II transcription factor binding
MED6
CSE2
ADR1
GAL11
GCN4
CYC8
YDR520C
SWI6
SRB2
SRB7
SSN2
SRB8
DAL81
Gene
Z_Shift_K_Gem
Z_lm_K_Gem
Z_Shift_K_Cyt
Z_lm_K_Cyt
Z_Shift_L_Gem
Z_lm_L_Gem
Z_Shift_L_Cyt
Z_lm_L_Cyt

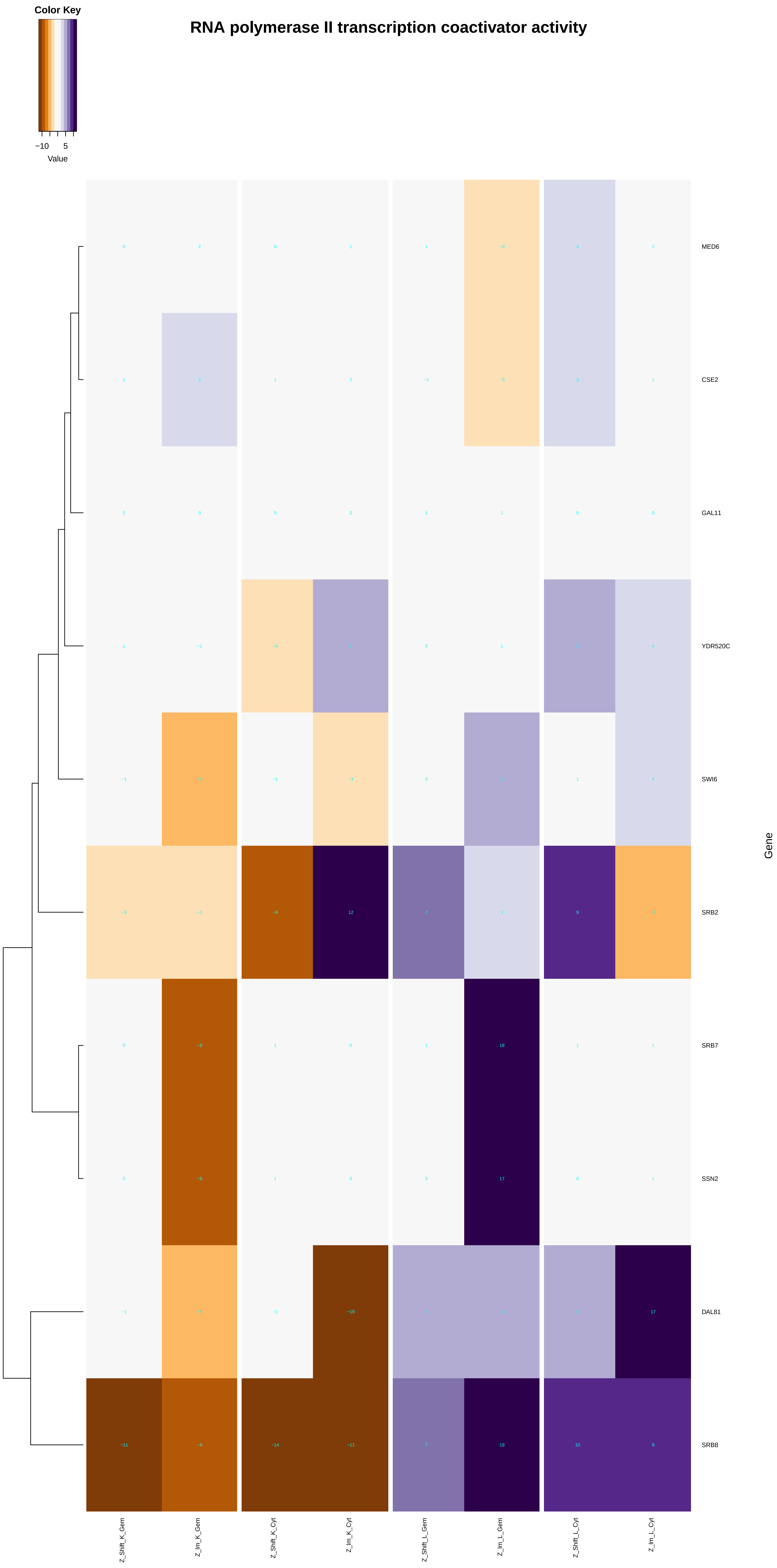

RNA polymerase II transcription coactivator activity
Color Key
−10
5
Value
MED6
CSE2
GAL11
YDR520C
SWI6
SRB2
SRB7
SSN2
DAL81
SRB8
Gene
Z_Shift_K_Gem
Z_Im_K_Gem
Z_Shift_K_Cyt
Z_Im_K_Cyt
Z_Shift_L_Gem
Z_Im_L_Gem
Z_Shift_L_Cyt
Z_Im_L_Cyt

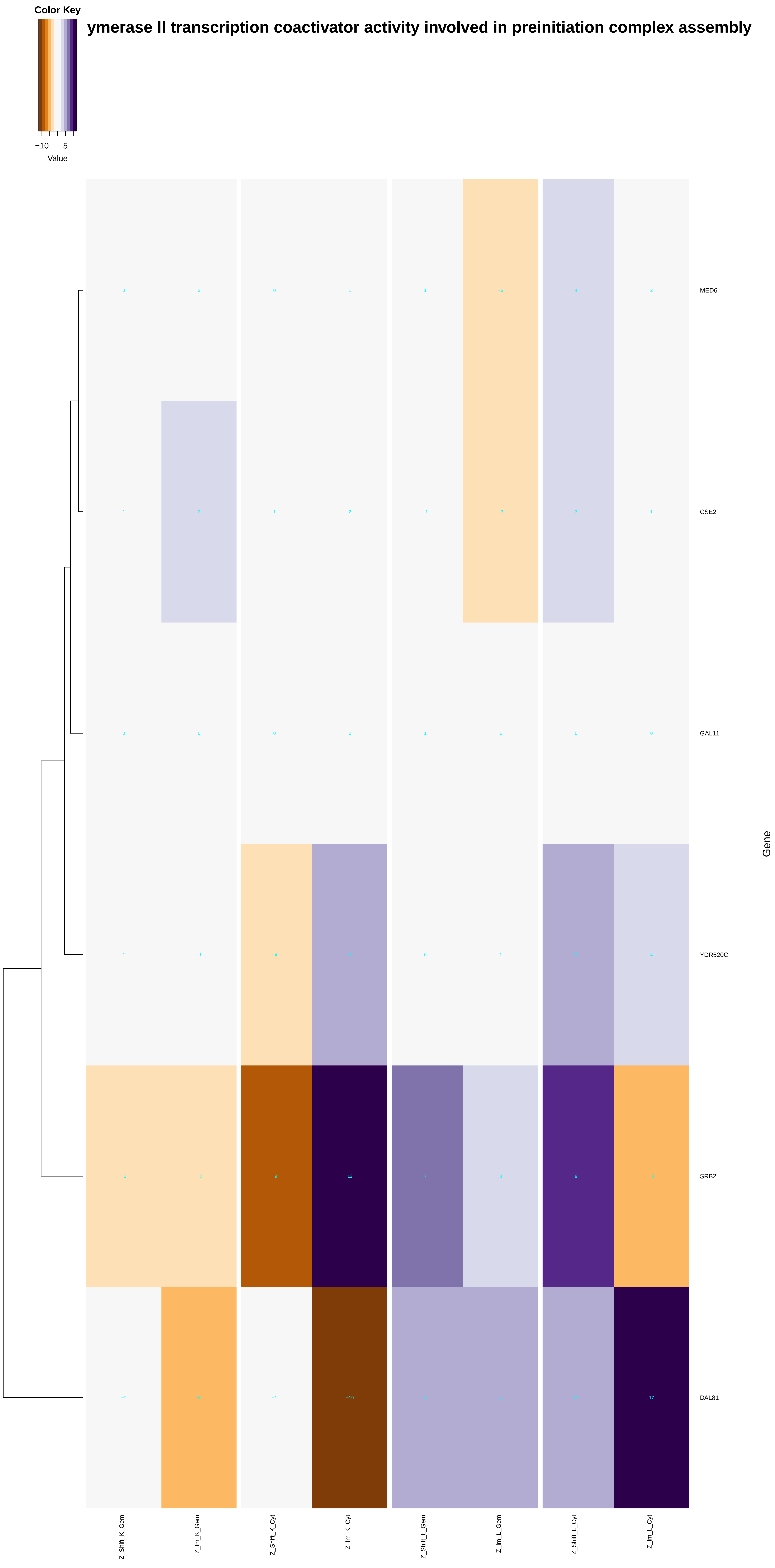

lymerase II transcription coactivator activity involved in preinitiation complex assembly
Color Key
−10
5
Value
MED6
CSE2
GAL11
YDR520C
SRB2
DAL81
Gene
Z_Shift_K_Gem
Z_Im_K_Gem
Z_Shift_K_Cyt
Z_Im_K_Cyt
Z_Shift_L_Gem
Z_Im_L_Gem
Z_Shift_L_Cyt
Z_Im_L_Cyt
